# Effectiveness of Alternating Heat and Cold Therapy on Pain and Labor Duration Among Primigravida: A Randomized Controlled Trial

**DOI:** 10.7759/cureus.93324

**Published:** 2025-09-27

**Authors:** Isabel Lawot, Imran Khan, Tumla Shrestha, Dinesh Kumar Bagga

**Affiliations:** 1 Department of Nursing, School of Nursing Science and Research, Sharda University, Greater Noida, IND; 2 Department of Midwifery, Maharajgunj Nursing Campus, Institute of Medicine, Tribhuvan University, Kathmandu, NPL; 3 Department of Medical-Surgical Nursing, School of Nursing Science and Research, Sharda University, Greater Noida, IND; 4 Department of Child Health Nursing, Maharajgunj Nursing Campus, Institute of Medicine, Tribhuvan University, Kathmandu, NPL; 5 Department of Orthodontics, School of Dental Sciences, Sharda University, Greater Noida, IND

**Keywords:** childbirth, cold heat therapy, duration of labor, labor pain, primigravida women

## Abstract

Background: Childbirth is often accompanied by significant discomfort and pain, making effective pain management an essential component of obstetric care. Due to the adverse effects of pharmacological methods, non-pharmacological options such as alternating heat and cold therapy are increasingly recognized for improving the birthing experience by reducing pain and shortening labor. This study aimed to assess the effect of alternating heat and cold therapy on labor pain and the duration of the active first stage of labor between experimental and control groups.

Methods: A randomized controlled trial was conducted among 106 primigravida admitted to Bharatpur Hospital, Chitwan, Nepal. Participants were randomly assigned to the intervention group (n = 53), who received alternating heat (38-40 ℃) and cold (0-5 ℃) water bags applied to the lower back, or to the control group (n = 53), who received standard care. Labor pain was measured using a visual analogue scale (VAS) at 4-5 cm, 7-8 cm, and 9-10 cm cervical dilation. Data were analyzed using descriptive and inferential statistics in SPSS.

Results: The mean age of participants was 24.23 ± 3.83 years in the intervention group and 23.15 ± 4.16 years in the control group. In the intervention group, mean labor pain scores decreased significantly from baseline to the last assessment (8.79 ± 1.09 to 6.77 ± 0.97, p < 0.001, Friedman test), whereas in the control group, pain increased slightly over time (8.51 ± 0.84 to 8.74 ± 0.65). The duration of the active first stage of labor was significantly shorter in the intervention group (5.09 ± 1.27 hours) compared to the control group (6.05 ± 1.79 hours; p = 0.012, t-test).

Conclusion: Alternating heat and cold therapy effectively reduced both pain intensity and the duration of labor in primigravida compared with standard care. With further validation, this approach may serve as a practical, non-pharmacological option for managing labor pain and duration.

## Introduction

Childbirth is an intense physiological and emotional experience, often accompanied by significant discomfort and pain, particularly during labor [1]. A study in Indonesia by Desmawati et al. reported that 97.6% of primiparous mothers perceived labor pain as severe [2]. According to Anim-Somuah et al., the intensity of labor pain and the coping strategies used by women can vary widely, with responses influenced by the circumstances and environment in which childbirth occurs [3]. Similarly, Unutkan and Yangın noted that fear of childbirth may lead to negative outcomes, including increased demand for pain relief, more medical interventions, prolonged labor, and dissatisfaction with the birthing experience. Providing support during childbirth has been shown to mitigate these adverse effects [4]. Besant and Maronge further emphasized that inadequate labor pain management can result in complications beyond physical discomfort, such as impaired ventilation, heightened anxiety and depression, and difficulties affecting breastfeeding and mother-infant bonding [5]. Various studies on non-pharmacological or complementary therapies for labor pain management have shown that these methods reduce the need for pharmacological interventions [6-8]. Thomson et al. reported that women who used analgesics sometimes experienced unwanted side effects, received less supportive care from healthcare providers, and felt guilt or inadequacy. In contrast, non-pharmacological methods, though not always effective in reducing pain or increasing the likelihood of spontaneous delivery, often encouraged women to remain engaged with their bodies and fostered a sense of teamwork with their birth partners [8]. Evidence also indicates that among nulliparous women, the mean duration of the active phase of labor ranges from 3.7 to 5.9 hours when non-pharmacological methods are applied [9]. Ariani found that cold therapy significantly reduced mean labor pain during the first stage of labor [10].

Beyond cold therapy alone, alternating heat and cold applications have also been reported as effective in managing labor pain and duration. Because this method poses minimal risk to both mother and baby, it is increasingly recognized as a safe and preferred non-pharmacological option for labor pain relief [11]. One study reported that the mean duration of labor following alternating heat and cold therapy was 6.72 ± 1.31 hours compared with 6.92 ± 0.94 hours in the control group, though the difference was not statistically significant [12]. Several studies have examined either heat or cold applications in relation to pain and labor progress. Building on this evidence, the present study aimed to evaluate the effect of alternating heat and cold applications on pain and labor duration among primigravida in both intervention and control groups.

## Materials and methods

Research design

In this study, a randomized controlled trial with a pre-test and post-test design was conducted to evaluate the effect of intermittent heat and cold therapy on pain and the duration of labor during the active first stage of childbirth. The study included primigravida admitted to the hospital maternity ward who had no pregnancy or labor complications and were expected to deliver normally.

Eligibility criteria

The inclusion criteria were primigravida aged 18-35 years, admitted to the maternity department of Bharatpur Hospital, Chitwan, Nepal, with no complications during pregnancy or labor. Participants were in the early stage of labor at 37-41 weeks of gestation, expected to have a normal delivery with a fetus of normal size confirmed by ultrasound, and willing to participate in the study.

Trial setting

The study was conducted in the maternity ward of Bharatpur Hospital, Chitwan, Nepal, a government hospital and maternal referral center in the central region of the country.

Ethical consideration

Ethical approval was obtained from the Nepal Health Research Council, Government of Nepal. The trial was registered at ClinicalTrials.gov (NCT06214585).

Intervention and comparator

Participants selected through simple random sampling were allocated to either the intervention (heat and cold) group or the control group. The researcher explained the alternating heat and cold therapy protocol to participants. The intervention was applied during the active first stage of labor, from 4-5 cm cervical dilation until full dilation. A warm compress, consisting of a hot water bag wrapped in a towel and maintained at 38-40 °C, was placed on the lower back for 30 minutes. This was followed by a cold compress, using a reusable ice pack wrapped in a towel and maintained at 0-5 °C, applied to the same area for 10 minutes. After a 30-minute rest period, the cycle was repeated and continued throughout the active phase of labor. The control group received routine hospital care provided to all women during childbirth.

Labor pain was assessed at baseline (4-5 cm dilation) and after intervention at 4-5 cm, 7-8 cm, and 9-10 cm cervical dilation. When full dilation (10 cm) was reached, the total duration of the active first stage of labor was recorded. Data collection was conducted between February and August 2024.

Outcomes measures

The primary outcome was labor pain, assessed using the visual analogue scale (VAS) at cervical dilations of 4-5 cm (before the intervention), immediately after the intervention, and again at 7-8 cm and 9-10 cm. At each stage, participants were asked to indicate their perceived pain level during uterine contraction relief. Another primary outcome was the duration of the active first stage of labor, measured from 4-5 cm cervical dilation until full dilation (10 cm).

Sample size of the study

The sample size was calculated using G*Power software (Heinrich Heine University, Düsseldorf, Germany). To achieve 80% statistical power with a 20% margin of error, 53 participants were required per group. The final sample included 106 participants, equally allocated between the intervention and control groups [11].

Randomization 

A total of 106 participants were selected through simple random sampling. Each day, women admitted to the maternity ward for normal delivery were screened for eligibility. Only those who were healthy, considered low risk, and provided informed consent were included. The process began by identifying the total number of women admitted that day from the admission register, after which their hospital identification numbers were retrieved from the main admission records.

**Figure 1:**
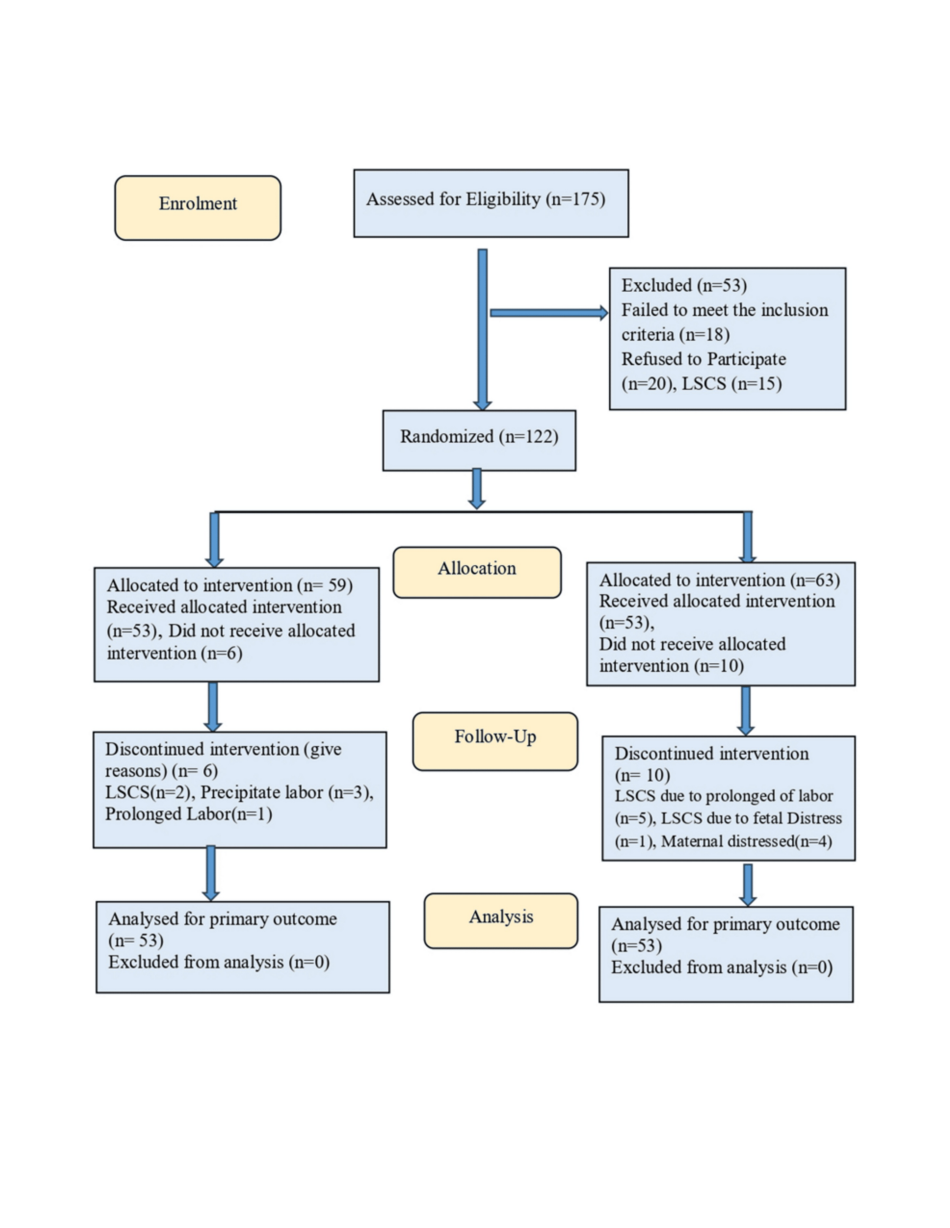
CONSORT diagram of the sample selection

Women who satisfied the set criteria were identified through their hospital registration numbers and considered for inclusion in the study. From this sampling frame, participants were chosen through simple random sampling using the hospital identification number. Sequence generation was performed by the principal investigator by shuffling these envelopes. Participants were assigned randomly to the study or control group by drawing numbered sealed envelopes by a co-investigator. Blinding was done during analysis. The selection of the sample is presented in the CONSORT [13] flow diagram (Figure 1).

Data analysis

Data were entered and analyzed using IBM SPSS Statistics for Windows, Version 16.0 (Released 2007; IBM Corp., Armonk, NY, USA). Descriptive and inferential statistics were applied according to the study objectives. Demographic characteristics were summarized using frequencies, percentages, means, and standard deviations. Differences between variables were examined using the Friedman test, the Wilcoxon signed-rank test, and an independent t-test.

## Results

Sociodemographic analysis of the 53 primigravida women in each group showed that in the experimental group, 39.6% were aged 22-25 years, while in the control group, the largest proportion (43.4%) were aged 18-21 years. The mean age was 24.23 ± 3.83 years in the experimental group and 23.15 ± 4.16 years in the control group. Most participants in both groups were Hindu (88.7% in the experimental group and 84.9% in the control group). The majority of women in the experimental group (77.4%) lived in joint families, whereas most in the control group (88.7%) lived in nuclear families. More than half of the participants in both groups (56.6% and 52.8%, respectively) were from rural areas (Table 1).

**Table 1:** Frequency and percentage distribution of sociodemographic variables among primigravida in the heat and cold and control groups

Sociodemographic variables		Heat and cold group, f (%)	Control group, f (%)
Age in years	18-21	13 (24.5)	23 (43.4)
	22-25	21 (39.6)	15 (28.3)
	26-30	16 (30.2)	12 (22.6)
	31-35	3 (5.7)	3 (5.7)
Religion	Hindu	47 (88.7)	45 (84.9)
	Others	6 (11.3)0	8 (15.1)
Type of family	Nuclear family	12 (22.6)	47 (88.7)
	Joint family	41 (77.4)	6 (11.3)
Residence	Urban	23 (43.4)	25 (47.2)
	Rural	30 (56.6)	28 (52.8)

The average pain scores in both the intervention (heat and cold) and control groups were similar before the application of alternating heat and cold therapy. After the intervention, pain scores in the experimental group began to decline and continued to decrease until the final assessment at 9-10 cm cervical dilation (6.77 ± 0.97). In contrast, pain scores in the control group did not decrease and instead remained high at the final assessment (8.74 ± 0.65) (Table 2). Analysis of pain levels in the experimental group showed a downward trend in mean ranks, indicating that alternating heat and cold therapy effectively reduced labor pain. The Friedman test confirmed this reduction as statistically significant (χ² = 75.55, p < 0.001). In the control group, no significant changes were observed, suggesting that without additional intervention, pain did not decrease (Table 2). Pairwise comparisons in the experimental group demonstrated that labor pain varied significantly across time intervals. Most comparisons showed a strong effect size, with statistically significant improvements from the pre-test to each post-test, reflecting a consistent and increasing treatment effect.

**Table 2:** Analysis of labor pain using Friedman and Wilcoxon signed-rank tests in the intervention and control groups *p < 0.05, **p < 0.001.

Test	Mean SD/mean rank	Heat and cold	Control group	Wilcoxon signed-rank (Z) (p-value)
Pre-test	Mean SD	8.79 ± 1.09	8.51 ± 0.84	Pre-test vs. post-test I (<0.001)**
	Mean rank	3.53	2.38	Pre-test vs. post-test II (<0.001)**
Post-test I	Mean SD	7.89 ± 1.09	8.51 ± 0.60	Pre-test vs. post-test III (<0.001)**
	Mean rank	2.61	2.34	Post-test I vs. post-test II
Post-test II	Mean SD	7.38 ± 0.74	8.58 ± 0.66	(0.006)*
	Mean rank	2.22	2.5	
Post-test III	Mean SD	6.77 ± 0.97	8.74 ± 0.65	Post-test I vs. post-test III
	Mean rank	1.64	2.78	(<0.001)**
Friedman test		75.55	6.17	Post-test II vs. post-test III
(χ^2^) p		<0.001 **	0.104	(<0.001)**

The mean duration of the first stage of labor was significantly shorter in the experimental group (5.09 ± 1.27 hours) compared with the control group (6.05 ± 1.79 hours). Independent t-test analysis confirmed that this difference was statistically significant, indicating that alternating heat and cold therapy effectively reduced labor duration (Table 3).

**Table 3:** Comparison of the duration of the first stage of labor between the heat and cold group and the control group among primigravida *p < 0.05.

Groups	Mean SD	95% CI	t-test
Heat and cold	5.09 ± 1.27	5.34-6.05	-2.56 (0.012)*
Control group	6.05 ± 1.79	5.84-6.82	

## Discussion

This study aimed to assess the impact of alternating warm compresses and ice pack applications on labor pain and the duration of the active first stage of labor among primigravida admitted to Bharatpur Hospital, Chitwan, Nepal.

The findings showed that the mean age of participants was 24.23 ± 3.83 years in the intervention group and 23.15 ± 4.16 years in the control group. Similar age patterns have been reported in other studies. For instance, Didevar et al. found mean ages of 21.87 ± 4.14, 22.29 ± 4.87, and 22.87 ± 5.23 years in the heat, cold, and non-intervention groups, respectively [14]. Likewise, Taavoni et al. reported mean ages of 24.43 ± 3.67 years in the experimental group and 24.80 ± 3.67 years in the control group, suggesting that most primigravida conceive within a similar age range [15].

In this study, the majority of primigravida women belonged to the Hindu community, with 88.7% in the intervention group and 84.9% in the control group. A similar trend was reported by Chaudhary et al. in India, where 81.3% of participants in the intervention group and 78.1% in the control group were Hindu [16]. This similarity may reflect the predominance of Hinduism in both countries.

Regarding family type, the majority of participants in both groups came from joint families, suggesting that women in such households may receive greater care and support during pregnancy. This finding contrasts with a study from Turkey, where most participants in both groups (62.8%) lived in nuclear families [17].

In this study, 56.6% of participants in the intervention group and 52.8% in the control group resided in rural areas. A comparable distribution (57.7%) was also reported in another study [18]. In contrast, a study conducted in India found that most participants lived in urban areas, with 78.4% in the intervention group and 62.2% in the control group [19].

The primary objective of this research was to evaluate the effect of alternating heat and cold therapy on labor pain, assessed using the VAS, and on the duration of the active first stage of labor, measured from cervical dilation of 4 cm to 10 cm.

The findings revealed that the mean labor pain scores were nearly identical in both groups during the pre-intervention period, with 8.79 ± 1.09 in the intervention group and 8.51 ± 0.84 in the control group. Sugandary et al. reported similar baseline findings in their study [11]. Following the first intervention, pain levels in the heat and cold group decreased slightly to 7.89 ± 1.09, while pain in the control group remained unchanged at 8.51 ± 0.60. Comparable results were reported in a study conducted in Spain [20].

By the second post-test, pain levels in the intervention group had decreased further to 7.38 ± 0.74, whereas the control group showed a continuous increase to 8.58 ± 0.66. A study by Devi et al. also reported similar reductions in labor pain with thermal interventions [21]. These results suggest that alternating heat and cold therapy during the active first stage of labor can help alleviate pain.

In the final post-test (III), the experimental group showed a significant reduction in pain levels (6.77 ± 0.97), while the control group continued to experience an increase (8.66 ± 0.65). Similar findings were reported in a study conducted in Turkey, where pain levels decreased in the experimental group but increased in the control group following cold therapy [22].

Comparison of pre- and post-test scores between the two groups using the Friedman test revealed a significant difference in the intervention group (χ² = 75.55, p < 0.001), but not in the control group (χ² = 6.17, p = 0.104). This indicates that alternating heat and cold therapy significantly reduced labor pain [11,22-24]. Post-hoc analysis with the Wilcoxon signed-rank test confirmed medium to large effect sizes and statistically significant improvements, consistent with findings from an Indian study that also observed significant differences across repeated assessments [11].

The overall duration of the active first stage of labor was shorter in the experimental group (5.09 ± 1.27 hours) compared to the control group (6.05 ± 1.76 hours), with a statistically significant difference. These findings are supported by evidence from multiple studies, several of which particularly emphasize the effectiveness of heat and cold therapy in reducing labor duration [11,22,24-26].

Limitations

The main limitation of this study was the inability to blind participants to the intervention due to its nature. In addition, the relatively small sample size limits the generalizability of the findings to broader populations and geographic regions.

## Conclusions

The study concluded that alternating heat and cold therapy significantly reduced labor pain compared to the control group, who showed no meaningful change over time. Additionally, participants who received the thermal intervention experienced a shorter duration of the active first stage of labor. However, these findings should be confirmed in larger-scale studies before this approach can be widely recommended for labor pain management and the reduction of labor duration.
